# In-depth analysis of alternative splicing landscape in multiple myeloma and potential role of dysregulated splicing factors

**DOI:** 10.1038/s41408-022-00759-6

**Published:** 2022-12-20

**Authors:** Anil Aktas Samur, Mariateresa Fulciniti, Herve Avet-Loiseau, Michael A. Lopez, Sanika Derebail, Jill Corre, Stephane Minvielle, Florence Magrangeas, Philippe Moreau, Kenneth C. Anderson, Giovanni Parmigiani, Mehmet K. Samur, Nikhil C. Munshi

**Affiliations:** 1 Department of Data Science, Dana Farber Cancer Institute, Boston, MA 02215 USA; 2 Department of Biostatistics, Harvard T.H. Chan School of Public Health Boston, Boston, MA 02115 USA; 3 Department of Medical Oncology, Dana Farber Cancer Institute, Harvard Medical School, Boston, MA 02115 USA; 4 University Cancer Center of Toulouse Institut National de la Santé, Toulouse, France; 5 Memorial Sloan Kettering Cancer Center, New York, 10065 USA; 6 Inserm UMR892, CNRS 6299, Université de Nantes; Centre Hospitalier Universitaire de Nantes, Unité Mixte de Genomique du Cancer, Nantes, France; 7 VA Boston Healthcare System, Boston, MA 02115 USA

**Keywords:** Cancer genomics, Myeloma

## Abstract

Splicing changes are common in cancer and are associated with dysregulated splicing factors. Here, we analyzed RNA-seq data from 323 newly diagnosed multiple myeloma (MM) patients and described the alternative splicing (AS) landscape. We observed a large number of splicing pattern changes in MM cells compared to normal plasma cells (NPC). The most common events were alterations of mutually exclusive exons and exon skipping. Most of these events were observed in the absence of overall changes in gene expression and often impacted the coding potential of the alternatively spliced genes. To understand the molecular mechanisms driving frequent aberrant AS, we investigated 115 splicing factors (SFs) and associated them with the AS events in MM. We observed that ~40% of SFs were dysregulated in MM cells compared to NPC and found a significant enrichment of SRSF1, SRSF9, and PCB1 binding motifs around AS events. Importantly, SRSF1 overexpression was linked with shorter survival in two independent MM datasets and was correlated with the number of AS events, impacting tumor cell proliferation. Together with the observation that MM cells are vulnerable to splicing inhibition, our results may lay the foundation for developing new therapeutic strategies for MM. We have developed a web portal that allows custom alternative splicing event queries by using gene symbols and visualizes AS events in MM and subgroups. Our portals can be accessed at http://rconnect.dfci.harvard.edu/mmsplicing/ and https://rconnect.dfci.harvard.edu/mmleafcutter/.

## Introduction

Alternative splicing (AS) processes a single mRNA precursor into one of the multiple transcript variants [1], resulting in isoform diversity that enhances proteome diversity and impacts a number of cellular processes [2, 3]. More than 60% of multiple-exon genes undergo AS [4], and many of those have cell type-specific isoforms. Abnormal splicing events are associated with malignant transformation [5, 6], and overall, splicing is altered in many human tumors and represents a unique vulnerability of cancer cells [7, 8]. Several AS changes recapitulate cancer-associated phenotypes by promoting angiogenesis, inducing cell proliferation, or avoiding apoptosis [9]. For instance, an AS event in exon 2 of Bcl-x results in two isoforms of Bcl-x with antagonistic effects on cell survival: Bcl-xL (long isoform), which is anti-apoptotic, and Bcl-xS (short isoform), which is pro-apoptotic [10]. Similarly, MCL1 is alternatively spliced by either skipping exon 2 to yield the pro-apoptotic MCL1S or including exon 2 to yield the anti-apoptotic MCL1L [11]. In addition to conferring an anti-apoptotic effect, AS can also modify genes to make cells resistant to therapy, as suggested for the CASP-2 gene among others [12].

Multiple myeloma (MM) is a highly heterogeneous disease driven by numerous genetic and epigenetic alterations and characterized by clonal proliferation of plasma cells in the bone marrow [3, 13, 14]. Aberrant splicing of individual genes has been implicated in the disease pathogenesis and response to therapy [15]. For example, splicing variation in *CRBN* transcripts is associated with acquired resistance to immunomodulatory drugs (IMiDs) [16]. Proteasome inhibitor therapy in MM leads to specific alterations in splice site usage and broad-scale interference with spliceosome function [17]. Recently, the impact of SF3B1 mutations on splicing patterns in MM was assessed, and differences between wild-type and mutated SF3B1 as well as a correlation between survival and the number of novel splice sites was found [18]. However, more generally, the molecular and cellular mechanisms driving aberrant AS events in MM cells compared to normal plasma cells (NPC) are poorly defined.

The discovery of the underlying causes and consequences of aberrant splicing is crucial to determining how these alterations contribute to the pathogenesis of MM. Splicing decisions depend on splicing factors (SFs) recognizing transcript-specific sequence elements. Therefore, point mutations in the transcripts or SFs as well as aberrant expression of SFs can lead to changes in splicing and provide a possible mechanism for increased cancer-cell dependency on splicing in the absence of driving mutations [19, 20]. Splicing factors like SRSF1, a member of the phylogenetically conserved serine/arginine-rich RNA binding protein family, are involved in a plethora of biological processes [21–23] and are functionally essential genes. For example, SRSF1 knockout is embryonically lethal [24], and it is upregulated in many malignancies even though the level of SRSF1 is tightly controlled within the cell [25–29].

Here, we investigated whether the global splicing landscape in MM cells is dysregulated compared to NPC. We evaluated the expression of splicing factors in a large dataset of MM patient samples and investigated the extent of AS events. We also investigated whether overexpression of SR (Serine/arginine) protein SRSF1 (previously known as SF2/ASF) drives aberrant AS in MM, thus impacting tumor growth and survival, which provides important biological insights with translational potential.

## Methods

### Patient Samples

We collected CD138+ MM cells from 323 newly diagnosed MM patients from the IFM/DFCI2009 clinical trial and NPCs from 16 donors. For quality control purposes, we confirmed plasma cell content (≥95%) following CD138+ selection by light chain staining. The median age of patients was 58 years (range: 30–65 years). Normal samples were collected from age-matched individuals with no known disease. Standard fluorescence in situ hybridization was performed on all patients to identify high-risk groups. All study participants provided written informed consent.

### RNA preparation and sequencing of the primary samples

After extracting RNA from each sample, RNA quantity and quality were evaluated using the Qubit RNA Assay Kit and Bioanalyzer using the RNA Pico Kit. Next, poly-A-selected library preparation for all newly diagnosed MM and normal plasma cell samples was done with the NEBNext Ultra RNA Library Prep Kit using ≥100 ng RNA per sample. Libraries passing QC were sequenced on the HiSeq 2000 system for 50 bp paired-end sequencing.

### Fluorescence in situ hybridization (FISH) analysis

Sorted plasma cells were fixed in Carnoy’s fixative and stored at −20 °C until hybridization. After slide preparation, they were denatured in 70% formamide for 5 min and dehydrated in a 70%, 85%, and 100% ethanol series. Probes specific for the t(4;14), del17p, and t(14;16) were purchased from Abbott Molecular and denatured separately for 5 min at 75 °C. After denaturation, the probes were dropped on the plasma cells and hybridized overnight at 37 °C. Then, coverslips were removed, and the slides were washed for 2 min in 2xSSC+0.1% Triton at 75 °C.

### RNA-seq quantification and splicing analysis

Paired-end 50 bp sequencing was performed for primary RNA samples and stranded 75 bp paired-end RNA sequencing for SRSF1 in-vitro samples. Raw files in fastq format were first evaluated with “FastQC v0.11.2” against any sequencing bias and error. All samples were evaluated after alignment with STAR (median reads per sample 79.3 M [32.2 M–168.6 M], >90% of samples had 50 M or more reads, Supplementary Table 3 for alignment QC), and only samples passing QC were kept for downstream analysis. The reference human genome (GRCh38) was downloaded from the GENCODE project website. RNA-seq samples in fastq format were aligned to the reference human genome using the STAR RNAseq aligner [30]. Samtools [31] was used to convert, sort, and index alignment files. Aligned data were analyzed using rMATS [32] 4.0.2 to identify differential AS events between: (1) MM and NPC; (2) OE and empty control for SRSF1 in MM cells; (3) KD and WT for SRSF1 in KMS11 and MM1S cell lines. Analyses were conducted for all five basic types of AS patterns using two biological replicates. rMATS uses a modified version of the generalized linear mixed model to detect differential AS events from RNA-seq data. We used the reads on target and junction counts (JCEC) for each AS event. All AS events were then filtered based on absolute Inclusion Level Difference (ΔPSI) > 0.1 between MM and NPCs with FDR < 0.05. The same filtering was used for SRSF1 cell lines. We ran rMAPS2 [33] (v2.0.0) using rMATS output for binding motif enrichment analysis, covering known binding motifs for RNA-binding proteins. Significantly spliced regions were used as the target regions for motif enrichment, and not significantly spliced regions were used for estimating background binding levels. We used 250 bp and 50 bp, respectively, as the length of the intronic and exonic regions to be examined and plotted. 50-bp sliding windows were used to count the motif occurrences, and the step size of windows sliding was set to 1. RSAT [34] was used to validate enrichment results from rMAPS. We converted the alternatively spliced regions identified with rMATS into a BED file and extracted hg38 sequenced using RSAT Sequence tools. FASTA files are generated with RSAT and then fed into matrix-scan (full options) using ATtRACT (2017) database. Homo Sapiens specific background model estimation method was used to correct estimations and results were reported as Enrichment of hits in the whole input sequence set. Change in the coding potential of the target genes after AS was estimated using Mapping Alternative Splicing Events to pRoteins (MASER) from the R/Bioconductor package. We used MASER’s “mapTranscriptsToEvents” function to identify transcripts potentially affected by alternative splicing events. We then annotated all transcripts attached to alternatively spliced regions using GTF files. Coding potential was considered switched only if gene type information in the GTF file for all new targets differs from all pre-splicing targets.

### Differential expression and pathways analysis

SRSF1 expression was compared between NPC, precursor conditions (MGUS, SMM), and newly diagnosed MM samples using the IFM 2009 [35], Mayo Clinic (GSE6477) [36], and Arkansas (GSE2654 and GSE5900) [37] datasets.

Transcript-level raw counts were estimated using the lightweight alignment tool Salmon [38] and converted to gene-level estimates by summing the estimated transcript counts. All genes with 0 counts were excluded from any downstream analysis before normalization. 26954 genes passed the filter and were used for normalization. Gene counts were normalized using DESeq2 [39], and differentially expressed genes were identified. The Reactome Functional Interaction network analysis in Cytoscape [40] was used for pathway analysis. Microarray datasets were downloaded from canEvolve.org [41] and analyzed using R and limma [42] packages.

### Visualization and statistical analysis

All downstream analyses were performed using R (v3.5.2). *DESeq2, limma*, and *edgeR* were used for normalization and differential gene expression analysis. *Pheatmap, UpsetR, rehape2, ggrepel, ggpubr, ggsignif, VennDiagram, survival, survcomp, readr*, *ggplot2, maser,* and *leafcutter* were used for visualization purposes. After selecting the top differentially expressed genes, expression data were scaled and clustered with the ward2 algorithm (ward. D2). All *p*-values are calculated with two-tailed tests.

#### Cell lines

KMS20 and MM1S cells were infected with pWZL-hygro retroviral vectors expressing T7-tagged SRSF1 cDNAs and selected with hygromycin for 72 h. The hairpin-containing PLKO.1 plasmid used for the generation of MM1S and KMS11 SRSF1-depleted cells was obtained from Sigma Mission. Packaged viral particles were used to infect MM cells using polybrene media (final concentration 8 µg/ml). Infected MM cells were selected by puromycin (0.5 µg/ml) for 48 h (Sigma, St. Louis, MO) and then left to recover for 24 h. CRISPR/Cas9 knockdown studies in the KMS11 MM cell line were performed using transEDIT lentiviral gRNA plus Cas9 expression vectors.

#### Cell proliferation and viability assays

The human MM cell lines (HMMCL) were cultured in RPMI 1640 (Mediatech, Herndon, VA) supplemented with 10% fetal bovine serum (FBS). MM cell growth was measured by a ^3^(H)thymidine (PerkinElmer, Boston, MA) incorporation assay. Cell viability was analyzed by CellTiter-Glo (CTG) (Promega). Statistical significance was determined by Student’s *t*-test.

#### Colony formation assays

KMS11 were seeded in a six-well tissue culture plate in semi-solid, methylcellulose media at a density of ~200 cells/well to enable single cells to proliferate into clonal populations. After 21 days, colonies were counted using an inverted microscope and gridded scoring dishes.

#### In vivo studies

KMS20 stably expressing wild-type SRSF1, or empty control were injected (8 × 10^6^ cells per site in 200 μl of phosphate-buffered saline) subcutaneously into the right flank of SCID mice. Mice were monitored for signs of distress and sacrificed after the animals showed at least four out of five signs of distress (ruffled fur, tremor, loss of mobility, kyphosis, anorexia).

#### Quantitative RT-PCR analysis

Expression of human SRSF1 transcript was determined using real-time quantitative reverse transcriptase–polymerase chain reaction (qPCR) based on TaqMan fluorescence methodology, following manufacturer protocols (Applied Biosystems, Foster City, CA). Relative expression was calculated using the comparative delta (Ct) method.

#### Western blotting

MM cells were harvested and lysed using RIPA lysis buffer. Cell lysates were subjected to sodium dodecyl sulfate-polyacrylamide gel electrophoresis SDS–PAGE, transferred to nitrocellulose membranes, and immunoblotted with SRSF1 and T7 antibodies (Thermo Fisher Cat#32-4500 and Sigma-Aldrich MABE50). Glyceraldehyde-3-phosphate dehydrogenase (GAPDH) or B-actin were used as a loading control (Santa Cruz Biotechnology).

## Results

### The landscape of alternative splicing changes in multiple myeloma

**Fig. 1:**
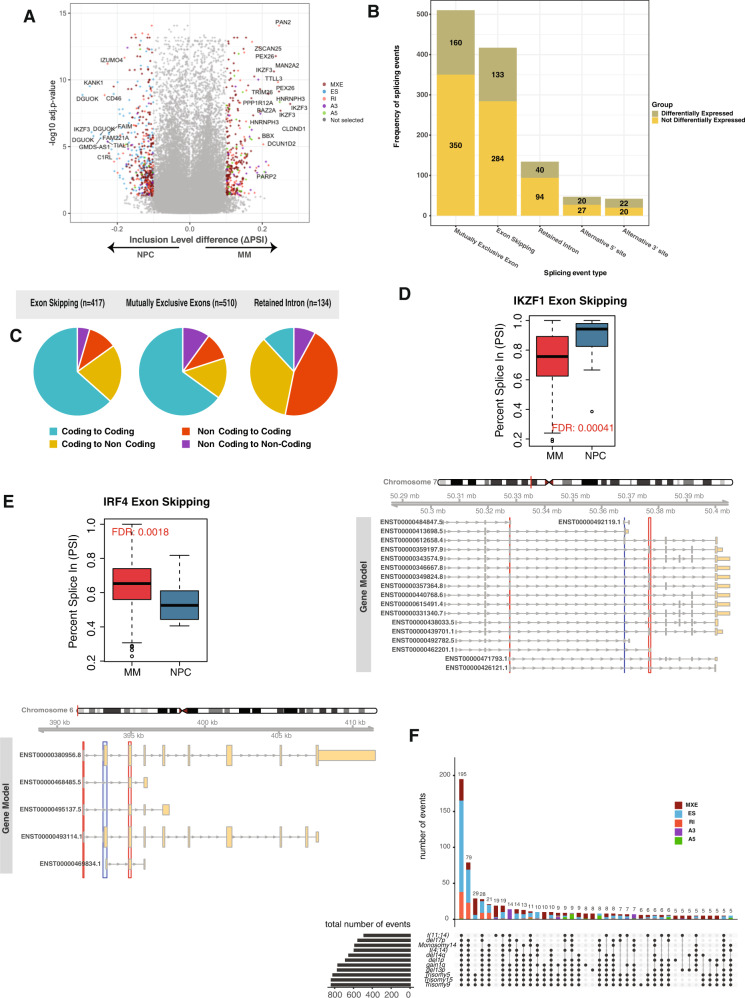
The landscape of alternative splicing (AS) events in multiple myeloma. **A** A volcano plot showing inclusion level (PSI) difference (*x*-axis) between MM (*n* = 323) and NPC (*n* = 16) and corresponding false discovery rate (FDR) (*y*-axis). Each point represents a splicing event. Events with ΔPSI > 0 (<0) indicate a higher (lower) utilization in MM compared to normal samples. Gray denotes splicing events that did not reach significance (adjusted *p* > 0.05 or −0.1 < ΔPSI < 0.1). Five splicing event types (MXE, ES, RI, A3, and A5) are indicated by different colors (red, blue, orange, purple, and green, respectively). **B** Number of splicing events (y-axis) by alternative splicing event type (x-axis). Splicing events occurring in genes differentially expressed between MM and normal samples are shown in green, others in yellow. **C** Distribution of transcripts by potential protein-coding ability change and AS event. Coding potentials are calculated by mapping splicing events to transcripts, as explained in the methods. The frequency of four categories (coding to coding, coding to non-coding, non-coding to coding, and non-coding to non-coding shown by four different colors) of possible changes is shown for ES, MXE, and RI, respectively. Green represents isoforms that remain protein-coding after AS, yellow for coding to non-coding switches, orange for non-coding to coding switches, and purple for isoforms that remain non-coding after AS. **D** Exon skipping event in *IKZF1*. MM cells skipped exon #5 more than NPC and tended to use a longer isoform. Boxplot in the upper panel shows the PSI levels for the skipped exon (marked with blue in the bottom panel). *IKZF1* isoforms and exons are shown in the bottom genomic track. Yellow thick boxes represent coding exons, and thin yellow boxes represent untranslated regions. Lines with arrows indicate the intronic regions and the strand where the represented gene is located. Red boxes show the upstream and downstream exons relative to spliced exons detected by rMATS; the blue box shows skipped exons. **E** Exon skipping event in *IRF4*. MM cells used exon #2 more than NPC. Boxplot in the upper panel shows the PSI levels for the skipped exon (marked with blue in the bottom panel). IRF4 isoforms and exons are shown in the bottom genomic track. Yellow thick boxes represent coding exons, and thin yellow boxes represent untranslated regions. Lines with arrows indicate the intronic regions and the strand where the represented gene is located. Red boxes show the upstream and downstream exons relative to spliced exons detected by rMATS; the blue box shows skipped exons. **F** Upset plot for the distribution of AS events by myeloma subgroup. The horizontal bar chart at the bottom left represents the total number of events found in each subgroup. The vertical bar chart in the top section indicates the number of event types shared by specific subgroups, with the membership combination indicated by gray circles in the center panel. Red indicates mutually exclusive exons (MXE), blue for exon skipping (ES), green for alternative 5 prime site (A5), purple for alternative 3 prime site (A3), and orange for retained intron (RI).

To assess AS differences, we analyzed deep RNA sequencing data from CD138 + MM cells isolated from 323 newly diagnosed patients and CD138 + NPC from 16 normal donors. In total, we identified 448,488 AS events between MM and NPC and computed the percent-spliced-in (PSI) scores. Using stringent criteria for AS between MM cells and NPC (FDR < 0.05 and absolute (ΔPSI) > 0.1), we identified 1150 differential AS events in 715 genes (Fig. 1A, Supplementary Fig. 1A). Global splicing analysis showed that events associated with exon usage alterations (mutually exclusive exons (MXE) (*n* = 510, 44.3%) and exon skipping (ES) (*n* = 417, 36.2%)) were the most frequent splicing events, while retained intron (RI) (*n* = 134, 11.6%), alternative 5’ site (A5) (*n* = 47, 4.0%), and alternative A3’ site (A3) (*n* = 42, 3.6%) were observed less frequently (Fig. 1B). Of these 1150 splicing events, 375 (33%) involved genes with significant differential expression (DE) between MM and NPC, whereas 775 (67%) occurred in genes not differentially expressed (Not-DE) (Fig. 1B). Alternative 3’ and 5’ site events were more common in DE genes than in Not-DE genes (46.1% vs. 31.3%, *p*-value = 0.003).

Some of the alternative splicing events imparted functional change by affecting the coding potential of the transcribed sequence. MXE and ES events retained the coding potential of most genes, with MXE and ES only changing the coding potential in 25% and 33% of the transcripts, respectively (Fig. 1C). RI altered 80% of the genes with a significant change from coding to non-coding or vice versa (Fig. 1C). A3SS and A5SS events altered the coding potential of 60% and 42% of their target genes, respectively (Supplementary Fig. 1B).

Using the DepMap CRISPR dataset (21Q1) [43, 44], we identified 154 (dependency score < −0.5 in at least 50% of MM cell lines) MM cell dependency genes with a significant alternative splicing event. These 154 genes constitute 21% of all MM dependency genes (*n* = 733). We then used a hypergeometric test to see if MM dependency genes are enriched in alternatively spliced genes (*n* = 715) and found a significant enrichment (hypergeometric test *p*-value 3.62e−19). MM dependency genes with significant splicing events included *MEF2C*, *IKZF3*, *IRF4*, *UBE2G2*, *PSMC1*, and *SMARCB1* (Supplementary Table 1). Overall, 20 tumor suppressors or oncogenes, 24 protein kinase genes, and 64 transcription factors were significantly differentially spliced between MM and NPC. These critical genes were affected by different splicing mechanisms. For example, compared to NPC, MM cells preferentially expressed a longer *IKZF1* transcript through alternative splicing involving DNA sequence recognition sites. Similarly, *IRF4* had altered DNA binding domains by alternatively utilizing the second exon. The utilization of these spliced products was significantly higher in MM cells than in NPC (Fig. 1D, E).

To evaluate the pathways and network patterns affected by the splicing alterations, we performed Reactome Functional Interaction network analysis using 715 alternatively spliced genes (Supplementary Table 1) and identified 15 protein-protein interaction networks using Reactome Protein Interaction database. In total, 148 unique pathways were enriched in 15 networks (Supplementary Fig. 2). Three clusters were enriched in pathways controlling transcription, splicing, and mRNA processes. One cluster was enriched in deubiquitination and proteasome pathways. Two clusters were enriched in B cell activation, B Cell receptor signaling, histone deacetylases, RXR, and RAR heterodimerization, which controls DNA binding (Supplementary Fig. 2).

Like mRNA levels, splicing patterns had significant alterations across MM subtypes (Fig. 1F). Splicing analysis comparing individual MM subtypes to NPC showed heterogeneity in the number and types of genes involved. Of 1150 events, 195 (17%) events were common to all MM subtypes (Fig. 1F, Supplementary Fig. 3, Supplementary Table 2), which affected 150 genes including *XBP1*, *SRSF7*, *IKZF3*, *STAT2*, *CD46*, and *IRF9*. Details about MM subgroups can be visualized using our portal.

### Dysregulated splicing factors in multiple myeloma

**Fig. 2:**
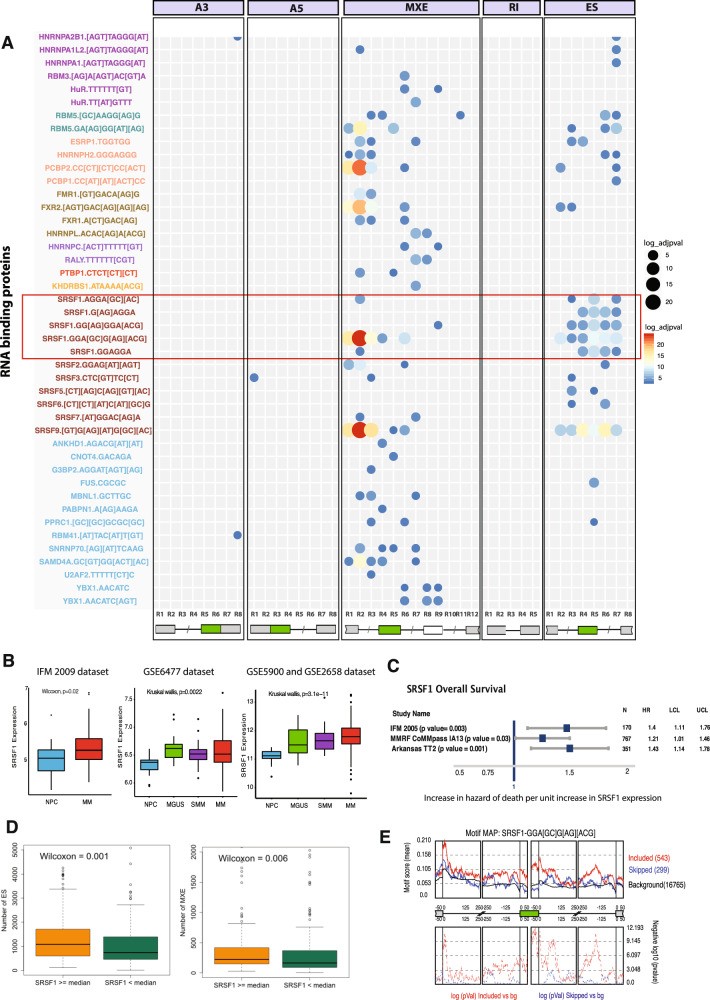
AS is Controlled by Splicing Factors (SFs). **A** SF motifs significantly enriched around AS events. The position (R) of each splicing event relative to the event type is represented in the *x* axis, 5’ to 3’. Splicing factors’ binding motifs are described in the *y* axis and were clustered using a sequence similarity network (SSN). Clustered SFs are shown in different colors. Each panel shows a single event type (A3, A5, MXE, ES, RI, respectively). The color and size of circles both indicate the significance of SFs. The red color and larger circles show the most significant SFs. Multiple regions were evaluated for each event type (bottom panel). Exons that are shown with green boxes are the region of interest for each event type. Exons represented with gray colors are up- and downstream exons. Lines separated with / are representing exons. Each region is numbered from R1 to R(n), where n is the total region for each event type. Intronic regions are 250-bp flanking sequences, and exon regions are 50-bp sequences from the start or end of the exon where splicing factors often bind. **B** Boxplots of SRSF1 expression (y axis) by MM stage (*x* axis) in three datasets (3 panels). Panel corresponds to datasets: IFM 2009 (left), Mayo Clinic-(GSE6477) (center), and Arkansas-(GSE5900 and GSE2658) (right). Colors correspond to MM (red), MGUS (green), SMM (purple), and NPC (blue). *p* values for each dataset are given on the top with corresponding test names. **C** Hazard ratio (*y* axis) shown with blue boxes and 95% CI (lines) of overall survival (OS) for SRSF1 expression in three datasets. p values were calculated using the cox proportional hazard model, and the summary table is on the right. **D** Boxplots of the number of exon skipping and MXE (*y* axis) events by SRSF1 expression (*x* axis). Green boxes show that patients with low SRSF1 expression have fewer splicing events (ES, MXE) compared to patients with high SRSF1 expression (orange boxes), as separated by the upper median. **E** Positional distribution of the SRSF1 binding motif. The middle section of the figure shows skipped exon (green box), 3’ end of the exon before (left gray box), and the 5’ end of the exon after (right gray box). Lines represent 250-bp intronic sequences before and after each shown exon. The top panel shows the mean motif score calculated as density within a 50-bp sliding window as the overall percentage of nucleotides covered by the SRSF1 binding motif. The black line indicates the background signal in this region. Red and blue lines show enrichment of SRSF1 binding motifs around exons more utilized by MM (red) or less utilized by MM cells (blue). The SRSF1 binding motif is shown above the panel. The panel at the bottom shows the -log10(p value) of the motif enrichment in these windows. The higher the peak reaches, the more significant the *p* value for that region. Significance was determined by comparison to a ‘background set’ of 16765 exons without splicing changes (rMATs FDR > 50%) in expressed genes (bottom section).

To further explore the role of AS in MM, we focused on understanding the molecular mechanisms driving aberrant AS. We identified a set of differentially regulated alternatively spliced regions in MM cells compared to NPC and performed motif enrichment analyses in the vicinity [33] of these alternatively spliced regions for 115 well-characterized RNA binding proteins. We observed RNA binding motifs (RBM) for 38 SFs, including multiple motifs from SRSF1, SRSF9, FXR2, PCBP2, and RBM5 (Fig. 2A and Supplementary Table 5 for RSAT results), which were significantly enriched around the regions alternatively spliced in MM cells. Among these five SFs, only the expression of SRSF1 was consistently upregulated in MM and its precursor conditions (MGUS and SMM) compared to NPCs (Fig. 2B). High SRSF1 expression was also significantly associated with shorter overall survival (OS) in three datasets (Fig. 2C). We further confirmed that patients with high SRSF1 expression also had significantly more ES and MXE (Fig. 2D). RBM analysis showed that ES events were typically accompanied by SRSF1 binding either within the exon skipped, or within 100 nucleotides upstream of the skipped 3′ splice site, whereas SRSF1‐mediated ES involved binding of SRSF1 within the downstream intron (Fig. 2E).

### SRSF1 promotes multiple myeloma cell growth

**Fig. 3:**
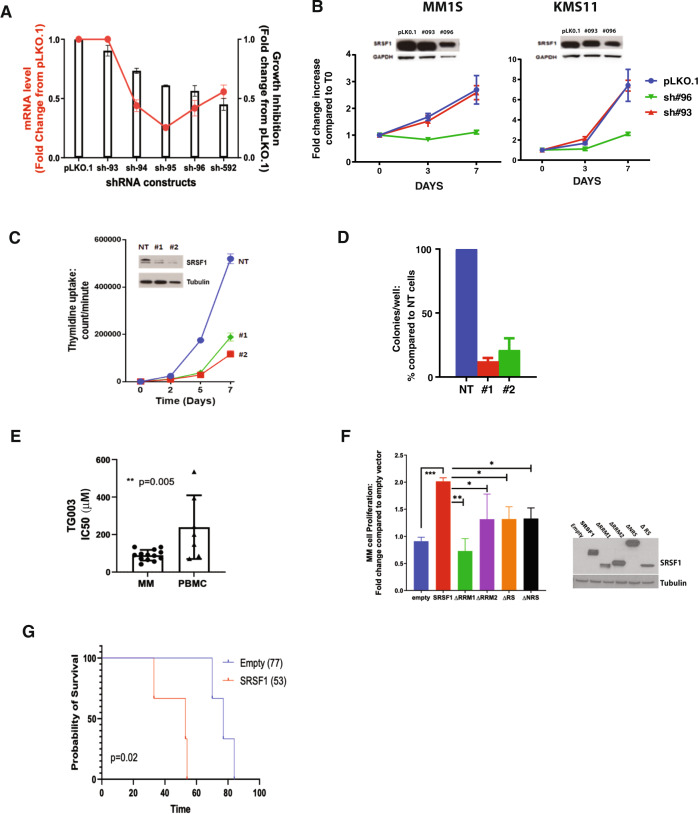
SRSF1 expression impacts MM cell growth and viability. **A** Genetic depletion of SRSF1 was achieved using four different shRNAs containing the target sequence or scrambled control in MM1S (*x*-axis). MM1S cells were infected with either scrambled (pLKO.1) or 4 SRSF1-targeted shRNAs and selected with puromycin for 72 h. qPCR analysis (right panel) was performed to confirm decreased SRSF1 mRNA levels (red line, right *y*-axis) in cells expressing SRSF1 shRNAs compared to scrambled cells (red line). Transduced cells were analyzed for effect on cell growth by 3(H) thymidine uptake and presented as fold change from cells infected with pLKO.1 (left *y*-axis and bar plots). Data are shown as the mean values ± s.d. of triplicates. **B** SRSF1 protein levels and cell proliferation were evaluated three days from puromycin selection by WB and CTG. Cellular proliferation is presented as the growth rate increase compared to *t* = 0. Reduced expression of SRSF1 is accompanied by a reduction of cell growth rate compared to control cells. **C** CRISPR/Cas9 knockdown studies were performed using transEDIT lentiviral gRNA plus Cas9 expression vectors in the KMS11 cell line. Cell growth was evaluated by 3H-thymidine uptake (*y* axis) over time (*x* axis). **D** CRISPR/Cas9 knockdown studies were performed using transEDIT lentiviral gRNA plus Cas9 expression vectors in the KMS11 cell line. CRISPR/Cas9 knockdown studies were performed using transEDIT lentiviral gRNA plus Cas9 expression vectors in the KMS11 cell line. Colony formation was measured in semi-solid, methylcellulose media. Graphs depict average colony numbers (mean ± SD) from control (NT) and KO MM cells in methylcellulose medium on day 21. **E** A panel of 13 MM cell lines (circles in left bar plot) and PHA-activated PBMC from seven healthy donors (triangles) were tested with different concentrations of TG003. IC50 analysis and a non-parametric t-test were performed. Data are shown as the mean value ± SD. **F** KMS20 MM cells were stably transduced with retroviruses expressing empty vector or T7-tagged SRSF1 mutants (*x*-axis). These cells were analyzed by western blotting with an anti-T7 antibody (right panel) to confirm transduction efficiency. Cell proliferation was evaluated after seven days of culture and represented as fold change from an empty control (*y*-axis). **G** SCID mice were injected with empty or SRSF1-expressing KMS20 cells (*n* = 3/cohort) and evaluated for overall survival. Log-rank Mantel-Cox test was utilized to assess statistical significance (*p* = 0.02).

To validate the role of SRSF1 in MM cell growth and survival, we first confirmed the protein expression of SRSF1 in a panel of MM cell lines and CD138+ primary cells (Supplementary Fig. 4A). We performed genetic loss-of-function (LOF) experiments by steady-state depletion of SRSF1 in MM cells using several specific shRNAs. We observed inhibition of cell growth (assessed by 3H-thymidine uptake; Fig. 3A and Supplementary Fig. 4B) and MM cell viability (measured by CellTiter-Glo; Fig. 3B) compared to scramble control as well as shRNA #93, which failed to KD SRSF1 protein. The impact of SRSF1 depletion on MM cell growth and colony formation potential was confirmed by CRISPR-Cas9 KO using two different guides targeting SRSF1 (Fig. 3C, D). These results are in agreement with the recently reported analysis of the CRISPR screen data in the DepMap [43, 44] (Avana library public 18Q448), which shows MM cell lines to be the most sensitive tumor cell type across >400 cancer cell lines to genetic ablation of core components of the U1-U2 spliceosome including SRSF1 [17].

Since inhibitors of RNA-binding protein kinases can regulate splicing by fine-tuning the phosphorylation of SR proteins [17], we also evaluated TG003, an inhibitor of Clk1/4, for its effect on MM cell proliferation. We indeed observed that TG003 decreased SRSF1 phosphorylation and induced a dose-dependent inhibition of MM cell viability, with a significant difference in the IC50 detected between MM cells and PHA-activated normal donor PBMCs (Fig. 3E and Supplementary Fig. 4C). We also observed high variance among PBMCs, highlighting a need for further evaluations of this cell type.

We further delineated the role of SRSF1 in promoting MM cell growth and survival by inducing ectopic expression of SRSF1 in the KMS20 cell line, which has low endogenous SRSF1 levels. Overexpression of SRSF1 led to a significant increase in MM cell proliferation compared to control cells in vitro (Fig. 3F) and impacted overall survival in vivo, where mice injected with SRSF1 overexpressing cells had a median survival of 53 days while mice injected with empty control cells had a median survival of 77 days (Fig. 3G).

The SRSF1 protein has a modular domain structure, with two RNA-recognition motifs (RRMs) as well as a serine/arginine-rich C-terminal domain (RS) for protein-protein interactions, subcellular localization, and recruitment of spliceosome components. To dissect the mechanisms involved in the SRSF1-induced growth effects, we also expressed SRSF1 mutants lacking either of the two RNA-recognition motifs (ΔRRM1 or ΔRRM2 mutants) or the RS domain (ΔRS mutant) in KMS20 MM cells. In contrast to full-length SRSF1, the SRSF1 mutants, which had an impaired ability to alter AS, were not as competent in inducing MM cell growth, suggesting an essential role for splicing regulation in SRSF1-mediated MM cell proliferation (Fig. 3F). These data were also confirmed in the MM1S MM cell line (Supplementary Fig. 4D).

Interestingly, we also observed that the expression of a chimeric SRSF1 protein harboring a nuclear retention signal (NRS) fused to its C-terminus (SRSF1-NRS1), which constitutively retains SRSF1 in the nucleus, failed to significantly promote MM cell proliferation (Fig. 3F). These results suggest that both nuclear (splicing) and cytosolic activities of SRSF1 have a role in promoting MM cell proliferation. SRSF1 indeed shuttles from the nucleus to the cytoplasm, where it participates in a wide range of post-splicing activities [19, 25, 45]. The evaluation of the SRSF1 interactome in MM cells expressing either full-length SRSF1 or SRSF1-NRS1 by mass spectrometry confirmed consistent interaction of nuclear SRSF1 with RNA-binding and processing proteins besides the normal components of the nucleus. In contrast, the cytosolic SRSF1 binds mostly to proteins involved in the translational process (Supplementary Fig. 4E).

**Fig. 4:**
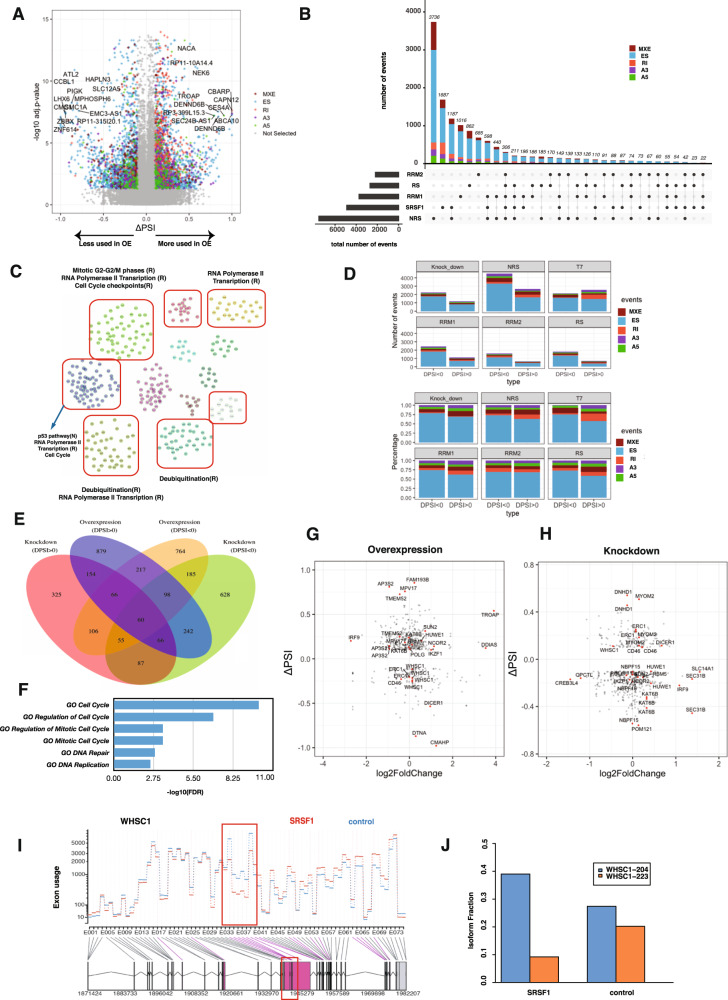
Analysis of splicing events regulated by SRSF1 modulation in MM cells. **A** Volcano plot showing inclusion level (PSI) change (*x*-axis) and false discovery rate (FDR) (*y* axis) between SRSF1 overexpressing and control samples in MM cell lines. Color-coded points on the right side shows spliced events that are more utilized in SRSF1 overexpressing cells (rMATS adjusted *p* < 0.05, ΔPSI > 0.1) while the converse (adjusted *p* < 0.05, ΔPSI > −0.1) holds in the left side. Gray denotes splicing events that are not significantly different (rMATS adjusted *p* > 0.05 or −0.1 < ΔPSI < 0.1) between SRSF1 overexpressing and control samples. Five splicing event types (MXE, ES, RI, A3, and A5) are shown with different colors (red, blue, orange, purple, and green, respectively). **B** Upset plot showing the number of AS events after SRSF1 overexpression. The horizontal bars at the bottom left represent the total number of events induced by each mutant type (black bars on the left). The vertical bars above indicate the number of events shared between mutant experiments. The color coding of splicing types is as in panel H. Shared events between mutant types are indicated with the connected black points and lines below the vertical bar chart. **C** The functional association analysis of spliced genes obtained by building a network using the Reactome functional interaction database. Genes are clustered (red boxes) by protein-protein interaction networks provided by Reactome FI with pathways assigned by their functions (blue arrows). **D** Total numbers (upper panel) and percentage (bottom panel) of SRSF1 regulated AS events, by type (color codes), in overexpressed (upper two rows) and knockdown (bottom two rows) samples compared to their respective control cells. **E** Venn diagram showing the overlap between SRSF1 overexpression and knockdown experiments for genes with significant alternative splicing events. **F** Pathways enriched for common gene targets identified from SRSF1 overexpression and knockdown experiments are shown in Fig. 2E. **G** ΔPSI (*x*-axis) and gene expression (log2 fold change) (*y* axis) change for each gene with significant splicing change after SRSF1 overexpression. **H** ΔPSI (*x*-axis) and gene expression (log2 fold change) (*y* axis) change for each gene with significant splicing change after SRSF1 knockdown. **I** Altered exon usage (*y* axis) of WHSC1 between SRSF1 overexpressing (red line) and control (blue line) samples. The gene model is represented on the *x* axis. The red box shows an alternatively spliced region in SRSF1 upregulated samples. **J** Isoform usage fractions (mean isoform utilization on *y* axis) of two isoforms (shown in blue and yellow) of *WHSC1* in SRSF1 overexpressing samples (left bars) and control (right bars).

To gain further insight into the role of SRSF1 in MM, LOF and gain-of-function (GOF) cells along with control cells were analyzed by RNA-seq to identify the change in genome-wide alternative splicing profiles regulated by SRSF1 in MM cells. We identified a total of 4888 (FDR < 0.05 and absolute (ΔPSI) > 0.1) SRSF1-regulated AS events in SRSF1 overexpressing MM cells compared to control cells (Fig. 4A). As expected, enforced SRSF1 nuclear retention (NRS) caused the highest number of AS events, with 3736 unique events (Fig. 4B, Supplementary Fig. 5A). Expression of SRSF1 mutants lacking either of the two RNA-recognition motifs (ΔRRM1 or ΔRRM2 mutants) or the RS domain (ΔRS mutant) caused fewer AS events (Fig. 4B, Supplementary Fig. 5B–D). Genes with AS events after enforced SRSF1 nuclear retention were enriched in the deubiquitination, cell cycle checkpoint, and mitotic G2-G2/M pathways (Fig. 4C). We further compared alternative splicing between patients with high and low SRSF1 expression (top 10% patients in each group) and performed gene set enrichment analysis. RNA Pol II transcription, spliceosome, and B cell receptors were common in this comparison (Supplementary Table 4). The majority of these AS events corresponded to ES (*n* = 3059) (Fig. 4A, D), with RI (*n* = 593) events as the second most common (Fig. 4A, D). SRSF1 promoted a similar number of exon inclusion and exclusion changes (Fig. 4A, B), either directly through RNA binding or indirectly through secondary interactions.

Analysis of AS events after SRSF1 KD in the KMS11 cell line revealed a total of 759 events (Supplementary Fig. 5E), with 348 genes (which is the union of 106 genes common between KD DPSI > 0 & OE DPSI < 0 and 242 genes common between KD DPSI < 0 & OE DPSI > 0) showing directionally consistent ΔPSI change between OE and KD experiments (Fig. 4E). These 348 genes were involved in the cell cycle, DNA repair, and DNA replication pathways (Fig. 4F). Some of these shared genes, including *WHSC1* (MMSET), *IRF9*, *DICER1*, *IKZF1*, *CD46*, and *ERC1*, are involved with MM pathobiology (Fig. 4G, H). For example, the overexpression of SRSF1 significantly changed the utilization of *WHSC1* exons (Fig. 4I), causing an isoform switch (Fig. 4J).

Next, we investigated the mechanism of SRSF1 upregulation in MM and observed that SRSF1 is amongst the top E2F1-regulated genes in MM1S (Supplementary Fig. 6A, B). Accordingly, E2F1 depletion decreased SRSF1 expression at the transcriptional and protein level (Supplementary Fig. 6C). Altogether, these data show that aberrant expression of an SF promotes MM cell growth and suggest that splicing modulation could be broadly effective across MM samples. Consistent with this and previous literature [17, 46], we observed a significant decrease in viability following exposure to SF3B1 inhibitor Pladeniolide B in a large panel of MM cell lines compared to PBMC from healthy donors (data not shown).

### Alternative splicing database for multiple myeloma

We have developed a R/Shiny-based web portal that allows the scientific community to access analysis results between MM, clinical subgroups, and NPC. A user-friendly interface can be accessed at http://rconnect.dfci.harvard.edu/mmsplicing for rMATS results and http://rconnect.dfci.harvard.edu/mmleafcutter for leafcutter results. Users can query splicing results generated by both tools using official gene symbols.

## Discussion

Splicing dysregulation is a major contributor to cancer phenotypes. Unlike previous studies focused on alternate splicing involving single genes such as Xbp-1 in MM, here we evaluated the global alternative splicing landscape using large-scale RNA-seq data from newly diagnosed patients with MM. We report profound and widespread abnormalities of AS in MM.

The alternative splicing events in MM frequently impacted the coding potential of the target genes, and the majority of the AS events did not involve differentially expressed genes. These two observations suggest that evaluating only gene expression without considering the actual isoform usage provides limited information about the transcriptomic alterations affecting disease biology. Our analysis also identified that certain events, such as SEs, are frequently shared amongst patients, while other events, such as MXE, are more individualized. This would suggest that specific AS events have tissue- and condition-specific behaviors, similar to non-coding RNAs. However, short RNA sequencing platforms and alternative splicing detection methods tend to bias towards finding ES and MXE events.

Further studies with long isoform sequencing platforms and more suitable techniques to detect broader event types without bias are needed to confirm these findings. Long isoform sequencing platforms like Oxford Nanopore would provide better resolution; however, their input sample requirements are usually much higher than Illumina-based methods, and it is often not possible to obtain enough plasma cells for every patient in large clinical studies. Although our study has a large RNAseq dataset, we would need to consider larger sets to measure individual variance in the future. In addition, alternative splicing tools can affect the interpretation of the results. Certain tools, such as rMATS, specifically use predefined splicing models and identify alternative splicing changes by comparing groups, making interpreting data easy. Extensions of these primary approaches, such as LeafCutter, jointly analyze groups of splice events and detect changes within each group [47] but make interpretation more challenging. We have also run LeafCutter on our dataset and added the results on our portal, where you can search the gene-level data (https://rconnect.dfci.harvard.edu/mmleafcutter/). The final comparison can be made on the isoform level. Techniques like isoform switch analysis utilize predicted isoform expression, and some try to explain them using AS events. Although the biological interpretation of these methods might be easier, they do not rely on direct evidence (reads covering the events). Bone marrow samples from normal donors are critical pieces in plasma cell cancers like MM. In our study, we used 16 samples; however, this caused a disproportional sample size that eventually affected the statistical power. Although this is the largest RNAseq dataset with normal plasma cells profiled similarly to MM cells, an increased normal sample pool would definitely help in future studies. Therefore, comparing the two conditions would benefit from increased sample size.

One of the key findings from our analysis was that specific treatment target genes, such as *IRF4* and *IKZF1*, had a significant alteration in splicing patterns compared to NPCs. This confirmed our previous data showing two separate isoforms of *IKZF1* [48]. This suggests the need to evaluate specific *IKZF1* isoforms in future studies to understand the impact of treatments like lenalidomide, for which IKZF1 is a primary target. Accordingly, therapeutic targeting of mis-splicing by small molecules may represent an innovative approach for treating MM, as shown by us and others.

Mutations in splicing machinery, such as SF3B1, are connected with splicing pattern changes in hematological cancers [18, 49]. However, mutations targeting splicing factors are very rare in MM. Similarly, other mutations in spliceosome components have also not been reported for any MM subtypes. The impact of these mutations should be addressed in further studies by inducing mutations in cell and animal models. While mutations in the core splicing machinery components drive dysregulated splicing in cancer, genome-wide splicing defects occur even in the absence of SF mutations. For instance, dysregulation of SF levels in cancer can arise from gene copy number or mRNA expression changes [19, 25, 45]. Recently, we reported miRNA-driven loops modulating the expression of SF and splicing modifiers (SM, enhancers/silencers) in MM patients [50]. Several studies prove that an abnormally expressed SF can have oncogenic properties by impacting alternative splicing of cancer-associated genes. We have identified all differentially expressed clinically relevant SFs and proteins involved in spliceosome assembly using a comprehensive evaluation of the genomic data from MM patient samples. This analysis provides the first look at the aberrations in splicing machinery impacting MM cell biology. MM subgroups driven by translocations or copy number changes showed significant overlap in our analysis, and unique splicing events in each of these subgroups were few. This may suggest that the spliceosome machinery regulating transcripts is similarly affected between subgroups. However, great heterogeneity among patients leaves the field open for further studies that may target various patient populations in lab environments.

Our motif enrichment analysis around alternatively spliced regions identified several splicing factors as the potential binding factors. This list includes several SRSF family members. We found higher expression of SRSF1 in MM patient cells, with a significant impact on clinical outcome. This information highlights that aberrant levels of “non-mutated” SFs provide a possible mechanism for increased dependency on splicing in the absence of driving mutations [19, 20]. Importantly, genetic modulation of SRSF1 in MM cells showed that altered expression of SRSF1 is associated with changes in AS and impacts MM cell proliferation. The mechanisms involved in the decreased MM cell growth after SRSF1 knockdown need further evaluation.

Nevertheless, this study represents an important example of SF facilitating tumor cell growth. Our results confirm that systematic targeting of SFs may perturb a distinct splice pattern, resulting in a better therapeutic index than global splicing inhibition [51, 52]. Our observation of E2F control of SRSF1 expression highlights the concept that splicing programs and transcriptional programs participate in the corruption of cellular processes during tumor initiation and progression. A similar observation was reported in breast cancer, where transcriptional upregulation of several spliceosome components, including SRSF1, resulted in MYC hyperactivation [45, 53] and oncogenic translation impacting critical SF networks [54]. In our study, we used sequence-based analysis; however, the targets we identified can be further validated and prioritized with other technologies like CLIP-seq. Future studies are needed to validate all these targets and their effects.

In conclusion, we report for the first time detailed genome-wide alternate splicing pattern changes in MM compared to NPCs. We examined the molecular and cellular mechanisms driving alternative RNA splicing in MM cells, providing evidence for the functional role and clinical significance of a gene in regulating alternative splicing in MM. These observations now provide the rationale for developing a therapeutic application to target spliced products and SFs in MM.

## Supplementary information

Supplementary Figure Legends

Supplementary Figure 1

Supplementary Figure 2

Supplementary Figure 3

Supplementary Figure 4

Supplementary Figure 5

Supplementary Figure 6

Supplementary Table 1

Supplementary Table 2

Supplementary Table 3

Supplementary Table 4

Supplementary Table 5

## Data Availability

Data generated and/or analyzed during the current study are available from the corresponding author.

## References

[1] Tress ML, Abascal F, Valencia A (2017). Alternative splicing may not be the key to proteome complexity. Trends Biochem Sci.

[2] David CJ, Manley JL (2010). Alternative pre-mRNA splicing regulation in cancer: pathways and programs unhinged. Genes Dev.

[3] Bolli N, Avet-Loiseau H, Wedge DC, Van Loo P, Alexandrov LB, Martincorena I (2014). Heterogeneity of genomic evolution and mutational profiles in multiple myeloma. Nat Commun.

[4] de Klerk E, t Hoen PA. Alternative mRNA transcription, processing, and translation: insights from RNA sequencing. Trends Genet. 2015;31:128–39. 10.1016/j.tig.2015.01.001 25648499

[5] Oltean S, Bates DO (2014). Hallmarks of alternative splicing in cancer. Oncogene..

[6] Song X, Wan X, Huang T, Zeng C, Sastry N, Wu B (2019). SRSF3-regulated RNA alternative splicing promotes glioblastoma tumorigenicity by affecting multiple cellular processes. Cancer Res.

[7] Dvinge H, Kim E, Abdel-Wahab O, Bradley RK (2016). RNA splicing factors as oncoproteins and tumour suppressors. Nat Rev Cancer.

[8] Visconte V, Makishima H, Jankowska A, Szpurka H, Traina F, Jerez A (2012). SF3B1, a splicing factor is frequently mutated in refractory anemia with ring sideroblasts. Leukemia..

[9] Climente-Gonzalez H, Porta-Pardo E, Godzik A, Eyras E (2017). The functional impact of alternative splicing in cancer. Cell Rep..

[10] Boise LH, Gonzalez-Garcia M, Postema CE, Ding L, Lindsten T, Turka LA (1993). bcl-x, a bcl-2-related gene that functions as a dominant regulator of apoptotic cell death. Cell..

[11] Bae J, Leo CP, Hsu SY, Hsueh AJ (2000). MCL-1S, a splicing variant of the antiapoptotic BCL-2 family member MCL-1, encodes a proapoptotic protein possessing only the BH3 domain. J Biol Chem.

[12] Droin N, Beauchemin M, Solary E, Bertrand R (2000). Identification of a caspase-2 isoform that behaves as an endogenous inhibitor of the caspase cascade. Cancer Res.

[13] Szalat R, Munshi NC (2015). Genomic heterogeneity in multiple myeloma. Curr Opin Genet Dev.

[14] Munshi NC, Avet-Loiseau H (2011). Genomics in multiple myeloma. Clin Cancer Res.

[15] Mimura N, Fulciniti M, Gorgun G, Tai YT, Cirstea D, Santo L (2012). Blockade of XBP1 splicing by inhibition of IRE1alpha is a promising therapeutic option in multiple myeloma. Blood..

[16] Gooding S, Ansari-Pour N, Towfic F, Ortiz Estevez M, Chamberlain PP, Tsai KT (2021). Multiple cereblon genetic changes are associated with acquired resistance to lenalidomide or pomalidomide in multiple myeloma. Blood..

[17] Huang HH, Ferguson ID, Thornton AM, Bastola P, Lam C, Lin YT (2020). Proteasome inhibitor-induced modulation reveals the spliceosome as a specific therapeutic vulnerability in multiple myeloma. Nat Commun.

[18] Bauer MA, Ashby C, Wardell C, Boyle EM, Ortiz M, Flynt E (2021). Differential RNA splicing as a potentially important driver mechanism in multiple myeloma. Haematologica..

[19] Paolella BR, Gibson WJ, Urbanski LM, Alberta JA, Zack TI, Bandopadhayay P, et al. Copy-number and gene dependency analysis reveals partial copy loss of wild-type SF3B1 as a novel cancer vulnerability. Elife. 2017;6:e23268. 10.7554/eLife.23268 PMC5357138 28177281

[20] Ge Y, Schuster MB, Pundhir S, Rapin N, Bagger FO, Sidiropoulos N (2019). The splicing factor RBM25 controls MYC activity in acute myeloid leukemia. Nat Commun.

[21] Mayeda A, Krainer AR (1992). Regulation of alternative pre-mRNA splicing by hnRNP A1 and splicing factor SF2. Cell..

[22] Karni R, Hippo Y, Lowe SW, Krainer AR (2008). The splicing-factor oncoprotein SF2/ASF activates mTORC1. Proc Natl Acad Sci USA.

[23] Li X, Manley JL (2005). Inactivation of the SR protein splicing factor ASF/SF2 results in genomic instability. Cell..

[24] Moroy T, Heyd F (2007). The impact of alternative splicing in vivo: mouse models show the way. RNA..

[25] Karni R, de Stanchina E, Lowe SW, Sinha R, Mu D, Krainer AR (2007). The gene encoding the splicing factor SF2/ASF is a proto-oncogene. Nat Struct Mol Biol.

[26] Anczukow O, Rosenberg AZ, Akerman M, Das S, Zhan L, Karni R (2012). The splicing factor SRSF1 regulates apoptosis and proliferation to promote mammary epithelial cell transformation. Nat Struct Mol Biol.

[27] Lee M, Dworkin AM, Gildea D, Trivedi NS, Program NCS, Moorhead GB (2014). RRP1B is a metastasis modifier that regulates the expression of alternative mRNA isoforms through interactions with SRSF1. Oncogene..

[28] Ezponda T, Pajares MJ, Agorreta J, Echeveste JI, Lopez-Picazo JM, Torre W (2010). The oncoprotein SF2/ASF promotes non-small cell lung cancer survival by enhancing survivin expression. Clin Cancer Res.

[29] Thorsen K, Mansilla F, Schepeler T, Oster B, Rasmussen MH, Dyrskjot L (2011). Alternative splicing of SLC39A14 in colorectal cancer is regulated by the Wnt pathway. Mol Cell Proteom.

[30] Dobin A, Davis CA, Schlesinger F, Drenkow J, Zaleski C, Jha S (2013). STAR: ultrafast universal RNA-seq aligner. Bioinformatics..

[31] Li H, Handsaker B, Wysoker A, Fennell T, Ruan J, Homer N (2009). The Sequence Alignment/Map format and SAMtools. Bioinformatics..

[32] Shen S, Park JW, Lu ZX, Lin L, Henry MD, Wu YN (2014). rMATS: robust and flexible detection of differential alternative splicing from replicate RNA-Seq data. Proc Natl Acad Sci USA.

[33] Hwang JY, Jung S, Kook TL, Rouchka EC, Bok J, Park JW (2020). rMAPS2: an update of the RNA map analysis and plotting server for alternative splicing regulation. Nucleic Acids Res.

[34] Santana-Garcia W, Castro-Mondragon JA, Padilla-Galvez M, Nguyen NTT, Elizondo-Salas A, Ksouri N, et al. RSAT 2022: regulatory sequence analysis tools. Nucleic Acids Res. 2022;11;50:W670–6. 10.1093/nar/gkac312 PMC9252783 35544234

[35] Samur MK, Minvielle S, Gulla A, Fulciniti M, Cleynen A, Aktas Samur A (2018). Long intergenic non-coding RNAs have an independent impact on survival in multiple myeloma. Leukemia..

[36] Chng WJ, Kumar S, Vanwier S, Ahmann G, Price-Troska T, Henderson K (2007). Molecular dissection of hyperdiploid multiple myeloma by gene expression profiling. Cancer Res.

[37] Zhan F, Barlogie B, Arzoumanian V, Huang Y, Williams DR, Hollmig K (2007). Gene-expression signature of benign monoclonal gammopathy evident in multiple myeloma is linked to good prognosis. Blood..

[38] Patro R, Duggal G, Love MI, Irizarry RA, Kingsford C (2017). Salmon provides fast and bias-aware quantification of transcript expression. Nat Methods.

[39] Love MI, Huber W, Anders S (2014). Moderated estimation of fold change and dispersion for RNA-seq data with DESeq2. Genome Biol.

[40] Shannon P, Markiel A, Ozier O, Baliga NS, Wang JT, Ramage D (2003). Cytoscape: a software environment for integrated models of biomolecular interaction networks. Genome Res.

[41] Samur MK, Yan Z, Wang X, Cao Q, Munshi NC, Li C (2013). canEvolve: a web portal for integrative oncogenomics. PLoS ONE.

[42] Ritchie ME, Phipson B, Wu D, Hu Y, Law CW, Shi W (2015). limma powers differential expression analyses for RNA-sequencing and microarray studies. Nucleic Acids Res.

[43] Meyers RM, Bryan JG, McFarland JM, Weir BA, Sizemore AE, Xu H (2017). Computational correction of copy number effect improves specificity of CRISPR-Cas9 essentiality screens in cancer cells. Nat Genet.

[44] Dempster JM, Rossen J, Kazachkova M, Pan J, Kugener G, Root DE, et al. Extracting Biological Insights from the Project Achilles Genome-Scale CRISPR Screens in Cancer Cell Lines. bioRxiv. 2019:720243.

[45] Koh CM, Bezzi M, Low DH, Ang WX, Teo SX, Gay FP (2015). MYC regulates the core pre-mRNA splicing machinery as an essential step in lymphomagenesis. Nature..

[46] Soncini D, Martinuzzi C, Becherini P, Gelli E, Ruberti S, Todoerti K (2022). Apoptosis reprogramming triggered by splicing inhibitors sensitizes multiple myeloma cells to Venetoclax treatment. Haematologica..

[47] Li YI, Knowles DA, Humphrey J, Barbeira AN, Dickinson SP, Kyung IMH, et al. LeafCutter: annotation-free quantification of RNA splicing. bioRxiv. 2017:044107. 10.1038/s41588-017-0004-9 PMC5742080 29229983

[48] Singh I, Lee SH, Sperling AS, Samur MK, Tai YT, Fulciniti M (2018). Widespread intronic polyadenylation diversifies immune cell transcriptomes. Nat Commun.

[49] Saez B, Walter MJ, Graubert TA (2017). Splicing factor gene mutations in hematologic malignancies. Blood..

[50] Adamia S, Abiatari I, Amin SB, Fulciniti M, Minvielle S, Li C (2020). The effects of MicroRNA deregulation on pre-RNA processing network in multiple myeloma. Leukemia..

[51] Papasaikas P, Tejedor JR, Vigevani L, Valcarcel J (2015). Functional splicing network reveals extensive regulatory potential of the core spliceosomal machinery. Mol Cell.

[52] Tejedor JR, Papasaikas P, Valcarcel J (2015). Genome-wide identification of Fas/CD95 alternative splicing regulators reveals links with iron homeostasis. Mol Cell.

[53] Anczukow O, Akerman M, Clery A, Wu J, Shen C, Shirole NH (2015). SRSF1-regulated alternative splicing in breast cancer. Mol Cell.

[54] Ciesla M, Ngoc PCT, Cordero E, Martinez AS, Morsing M, Muthukumar S (2021). Oncogenic translation directs spliceosome dynamics revealing an integral role for SF3A3 in breast cancer. Mol Cell.

