# Supplementary material for: In-depth analysis of alternative splicing landscape in multiple myeloma and potential role of dysregulated splicing factors
Source: Blood Cancer J. 2022 Dec 20;12(12):171. doi: 10.1038/s41408-022-00759-6 (PMC9763261; doi:10.1038/s41408-022-00759-6)
Supplement: Supplementary file 1 — Supplementary Figure Legends [file 41408_2022_759_MOESM1_ESM.docx]

**Supplementary Figures Legends**

**Supplementary Figure 1. Splicing Events in Multiple Myeloma**

1. Inclusion level (PSI) difference (x-axis) between MM (n=323) and NPC (n=16) and corresponding false discovery rate (FDR) (y-axis). Each point represents a splicing event. Events with ΔPSI>0 (<0) indicate a higher (lower) utilization in MM compared to Normal samples. Red points (dark red is differentially expressed and light red is not differentially expressed) and blue points (dark blue is differentially expressed, light blue is not differentially expressed) are showing significantly spliced genes (adjusted p value < 0.05 and absolute(ΔPSI) >= 0.1). Grey denotes splicing events that we do not consider significantly different between the two groups (adjusted p value > 0.05 or absolute(ΔPSI) < 0.1).
2. Distribution of transcripts by potential protein-coding ability change for alternative 3’ and alternative 5’ usage events. Green represents isoforms that remain protein-coding after AS, yellow coding to non-coding switches, orange non-coding to coding switches, and purple color represents isoforms that remain non-coding after AS.

**Supplementary Figure 2. Pathway analysis of spliced genes based on functional protein networks**

Alternatively spliced genes were clustered based on the Reactome protein-protein interaction database (red boxes), and gene set enrichment analysis was performed for Reactome and KEGG terms (blue arrows).

**Supplementary Figure 3. Distribution of ASEs in myeloma subgroups**

The horizontal bars on each panel’s left side represent the total number of events in each subgroup. The vertical bars on top of each panel indicate the number of shared events between subgroups. Subgroups sharing the events are shown with connected black points below the vertical bars. A) Exon Skipping, B) Mutually Exclusive Exons, C) Retained Intron, D) Alternative 3’ site, E) Alternative 5’ site

**Supplementary Figure 4.**

1. Protein lysates from a panel of MM cell lines, primary MM cells from patients, and healthy donors' blood cells were analyzed for SRSF1 expression by western blot. GAPDH was used as the loading control.
2. MM cells growth inhibition after SRSF1 knockdown using shRNA compared to control sample.
3. RPMI-8226 cells were treated with several doses of TG003 for 24hours. Western blot analysis was performed to evaluate the expression of pSRSF1 and SRSF1.
4. A panel of 10 MM cell lines was tested for pSRSF1 expression by western blot. Individual TG003 IC50 was correlated with the level of pSRSF1.
5. MM cell growth increase in MM1S cell line with different SRSF1 mutants.
6. Enrichment analysis for nuclear and cytoplasmic proteins binding to SRSF1 is identified by mass-spec analysis.

**Supplementary Figure 5. Splicing changes for overexpressed and knockdown**

Volcano plot showing inclusion level (PSI) change (x-axis) and false discovery rate (FDR) (y-axis) between mutant overexpressing and control samples in MM cell lines. Color-coded points on the right side shows spliced events that are more utilized in SRSF1 overexpressed cells (adjusted p < 0.05, ΔPSI >0.1) while the converse (adjusted p < 0.05, ΔPSI > -0.1) holds in the left side. Grey denotes splicing events that are not significantly different (adjusted p >0.05 or -0.1 < ΔPSI < 0.1) between SRSF1 overexpressing and control samples. Five splicing event types (MXE, ES, RI, A3, A5) are shown with different colors (red, blue, orange, purple, green, respectively). A) NRS, B) RRM1, C) RRM2, D) RS, E) Knockdown

**Supplementary Figure 6.**

1. E2F1 and DP1 binding on SRSF1 promoter in MM1S cell line identified by ChIP-seq.
2. ChIP was performed using E2F1 Ab or control IgG followed by quantitative PCR in MM1S and CD138+ MM cells. Data represented as fold enrichment over input.
3. Correlation between SRSF1 and E2F1 expression detected by qPCR in 14 samples
4. MM1S MM cell line was treated with RK-19 peptide for 24 hours to disrupt DP1/E2F1 heterodimerization, and SRSF1 expression was evaluated by WB using Tubulin as the loading control.

**Supplementary Table 1. Alternatively spliced genes and DEPMAP score.** Alternatively spliced 715 genes are given in rows and event type, gene symbols, median DEPMAP score and dependency in MM cell line are given in columns.

**Supplementary Table 2.**  List of alternatively spliced genes and event types in MM subgroups.

**Supplementary Table 3.** Alignment QC measurements for each sample in the study reported by STAR RNAseq aligner.

**Supplementary Table 4.** Gene set enrichment analysis results for alternatively spliced regions between SRSF1 high and SRSF1 low patients.

**Supplementary Table 5.** RSAT enrichment results for splicing factor enrichment in alternatively spliced regions.
