# Supplementary figures and images for: In-depth analysis of alternative splicing landscape in multiple myeloma and potential role of dysregulated splicing factors
Source: Blood Cancer J. 2022 Dec 20;12(12):171. doi: 10.1038/s41408-022-00759-6 (PMC9763261; doi:10.1038/s41408-022-00759-6)

## Supplementary Figure 1

**A**

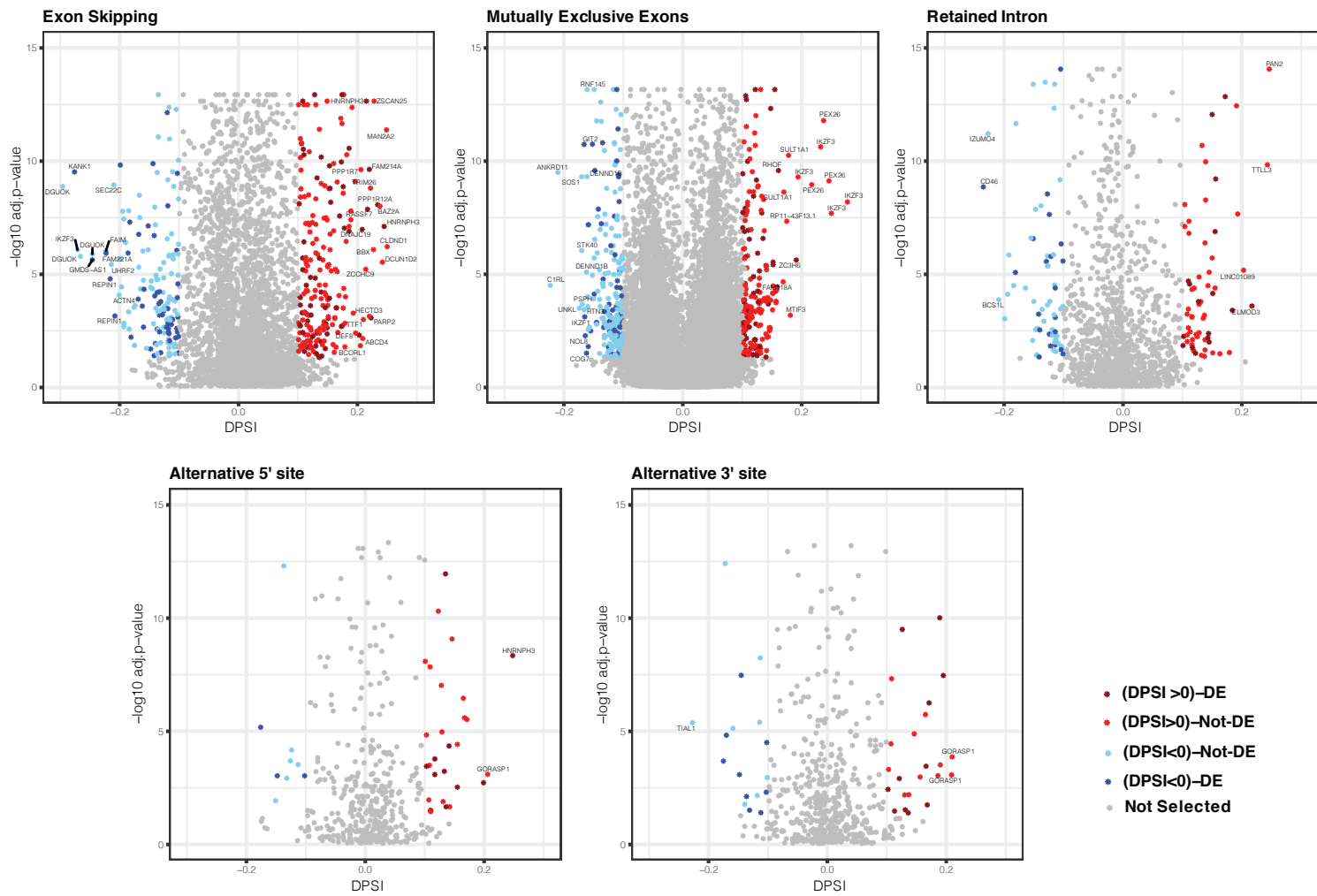

# B

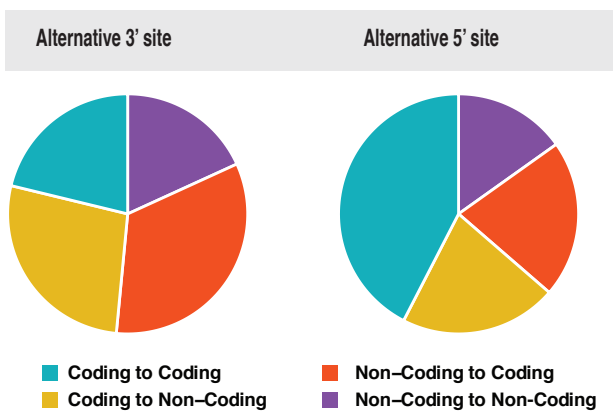

Supplement: Supplementary file 2 — Supplementary Figure 1 [file 41408_2022_759_MOESM2_ESM.pdf]

## Supplementary Figure 2

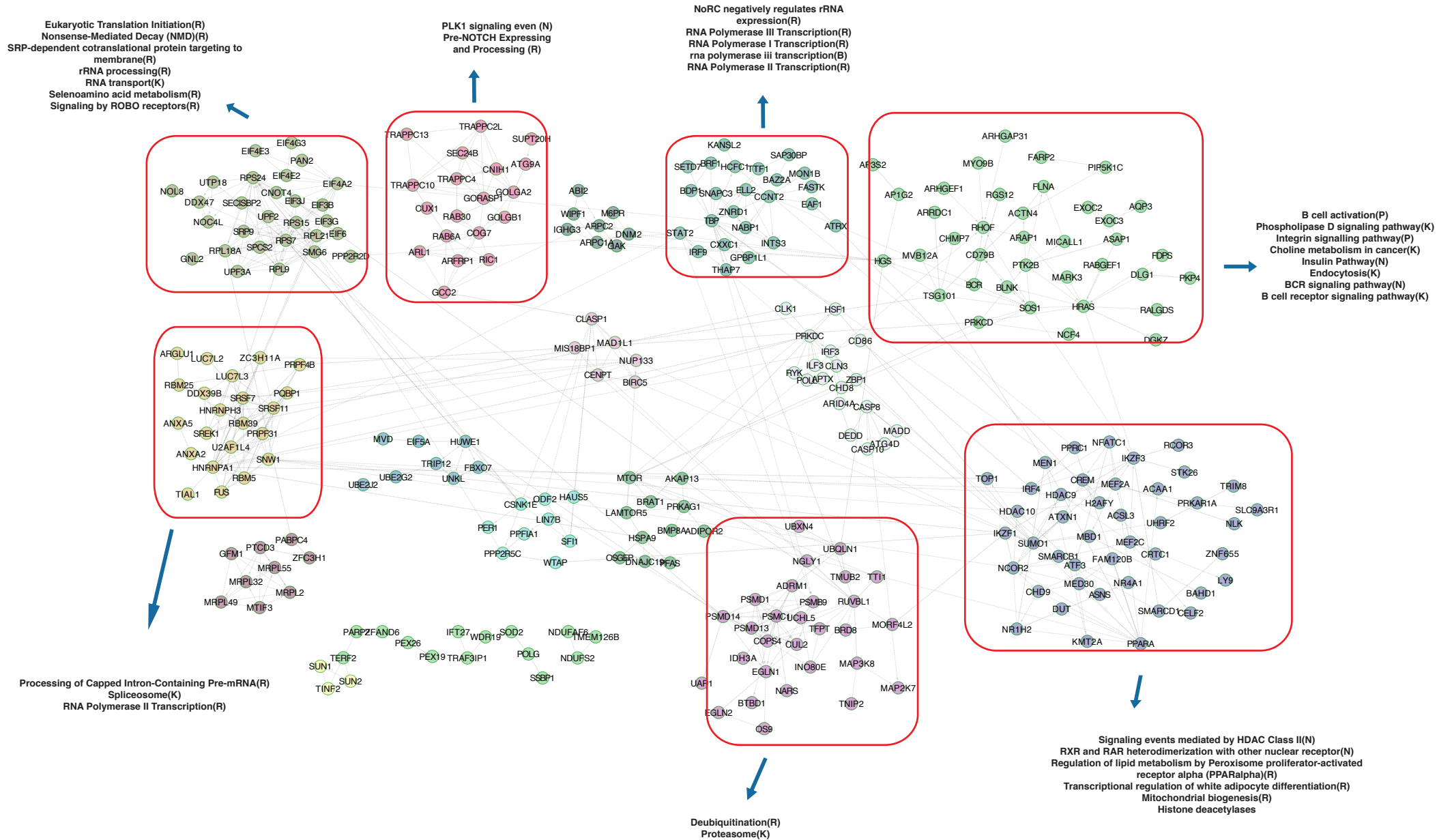

Supplement: Supplementary file 3 — Supplementary Figure 2 [file 41408_2022_759_MOESM3_ESM.pdf]

# A

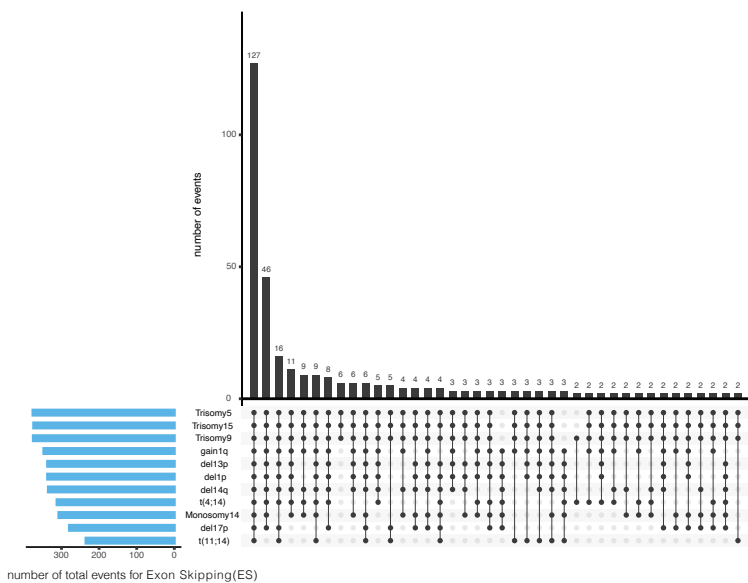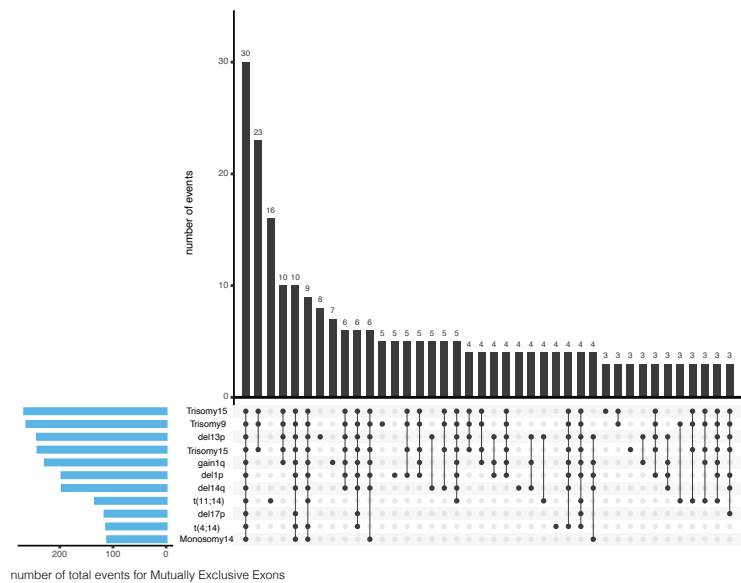

**C**

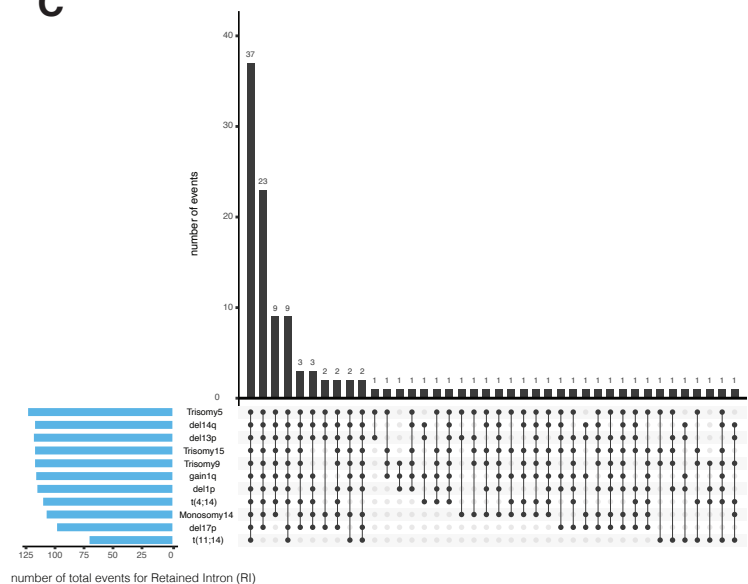

## D

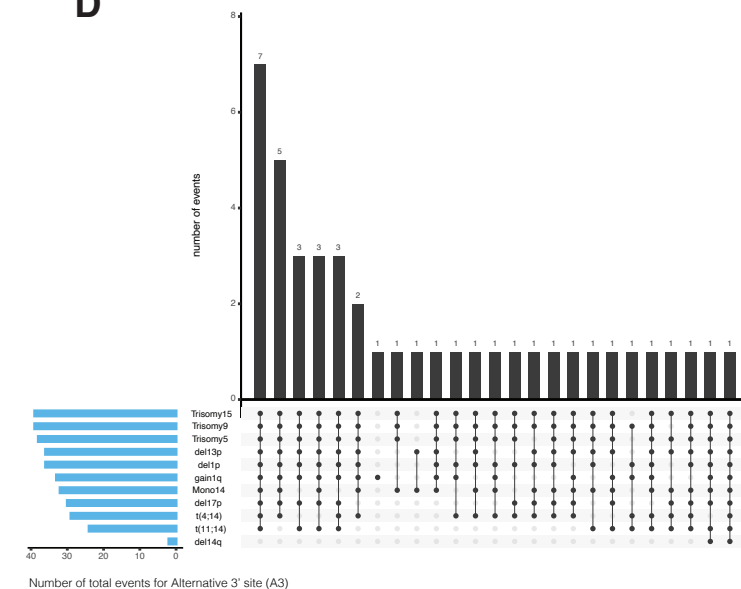

# E

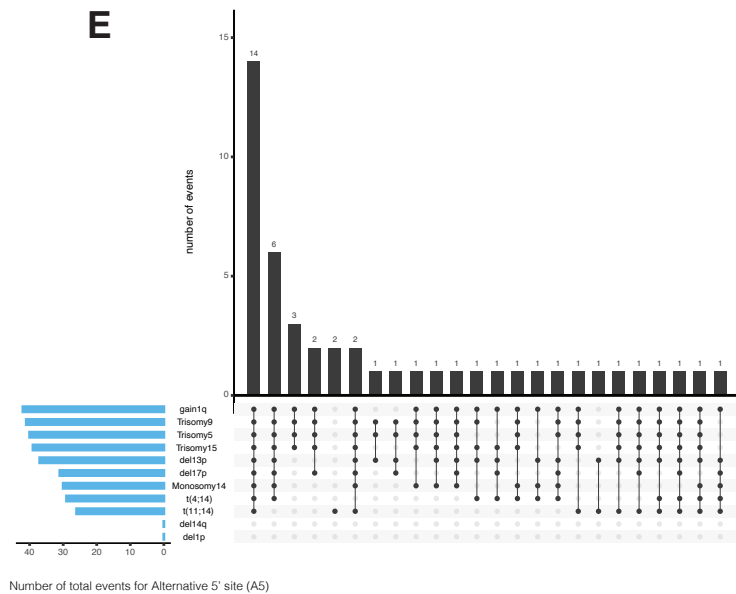

Supplement: Supplementary file 4 — Supplementary Figure 3 [file 41408_2022_759_MOESM4_ESM.pdf]

Supplementary Figure 4

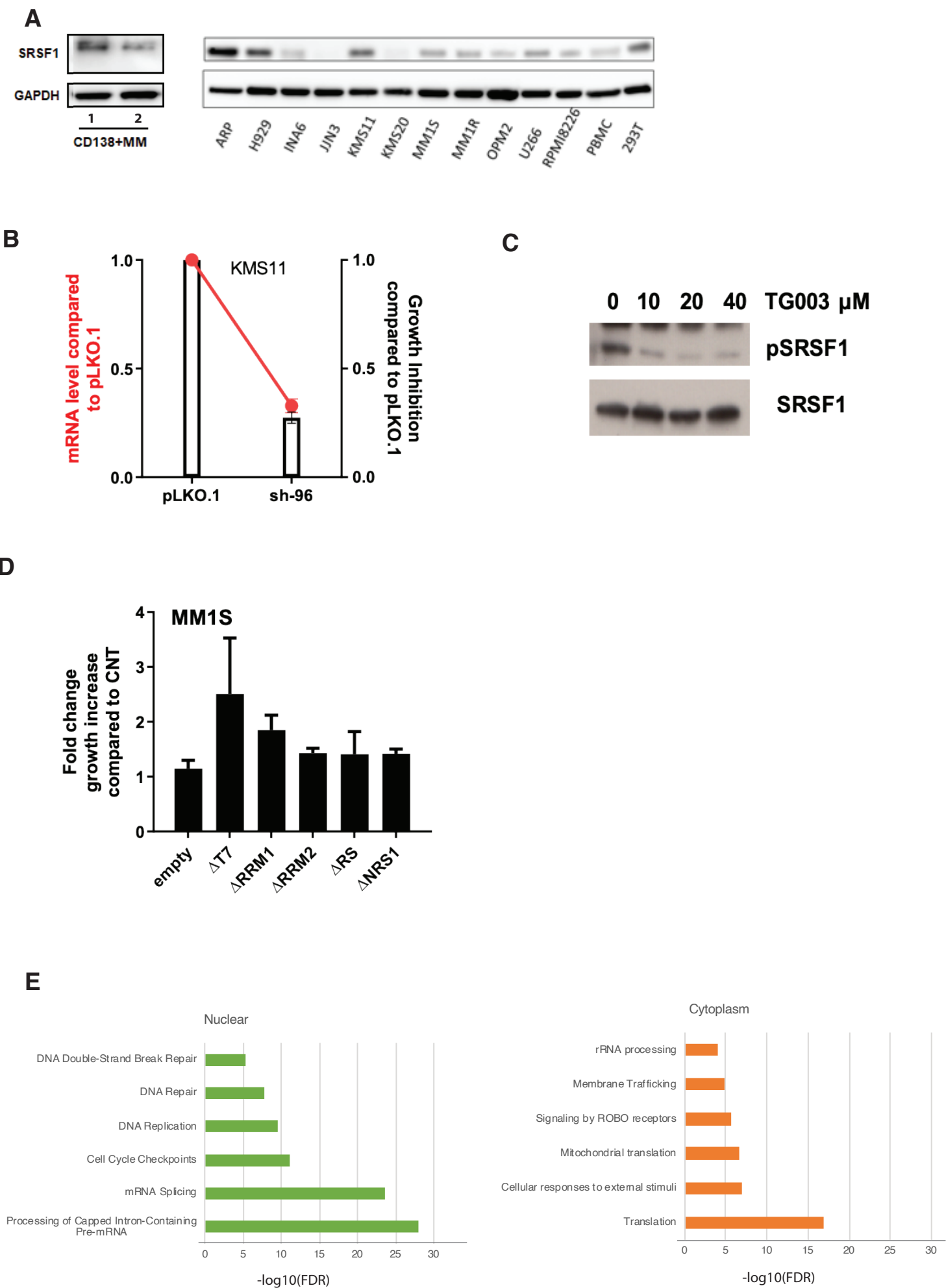

Supplement: Supplementary file 5 — Supplementary Figure 4 [file 41408_2022_759_MOESM5_ESM.pdf]

Supplementary Figure 5

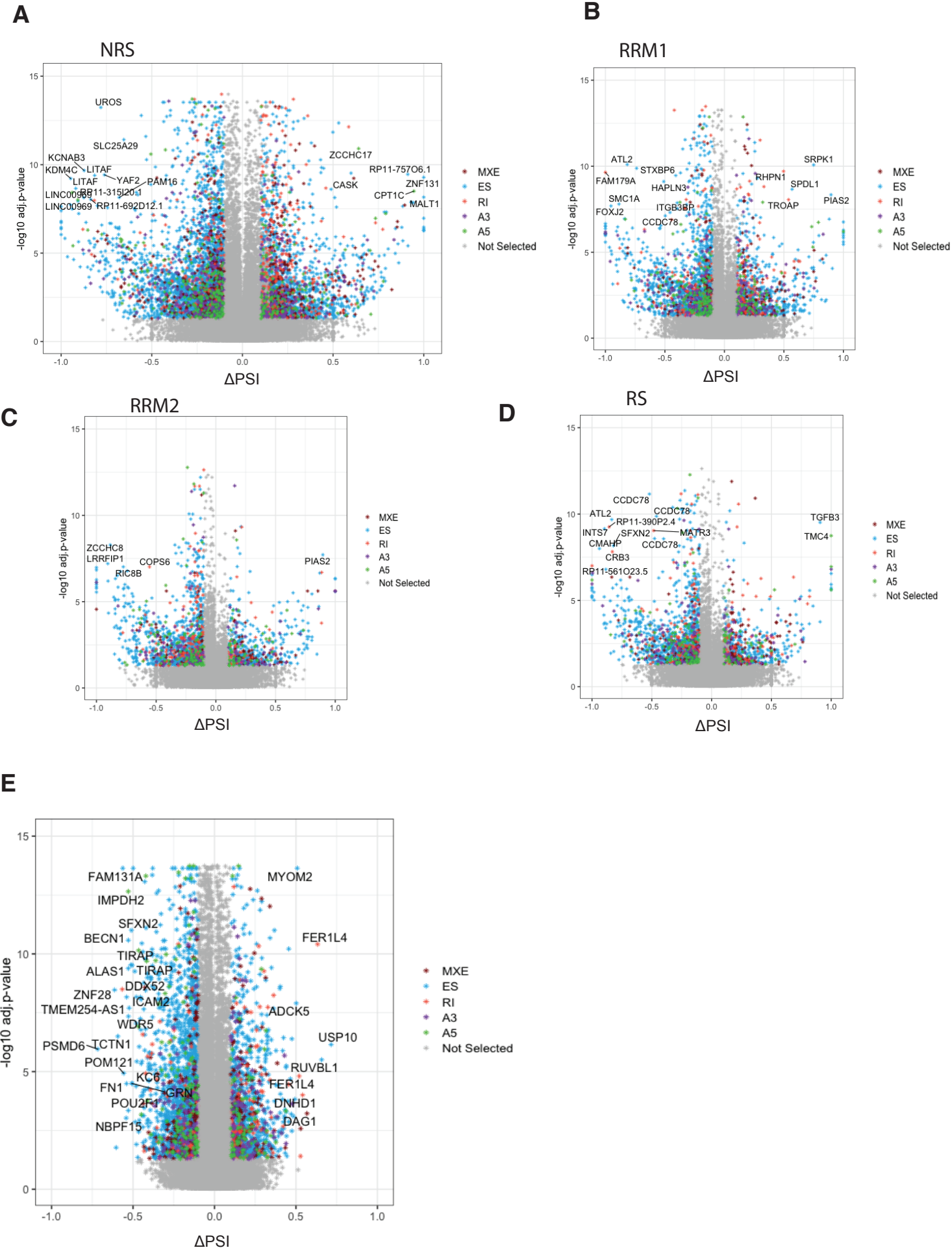

Supplement: Supplementary file 6 — Supplementary Figure 5 [file 41408_2022_759_MOESM6_ESM.pdf]

Supplementary Figure 6

A

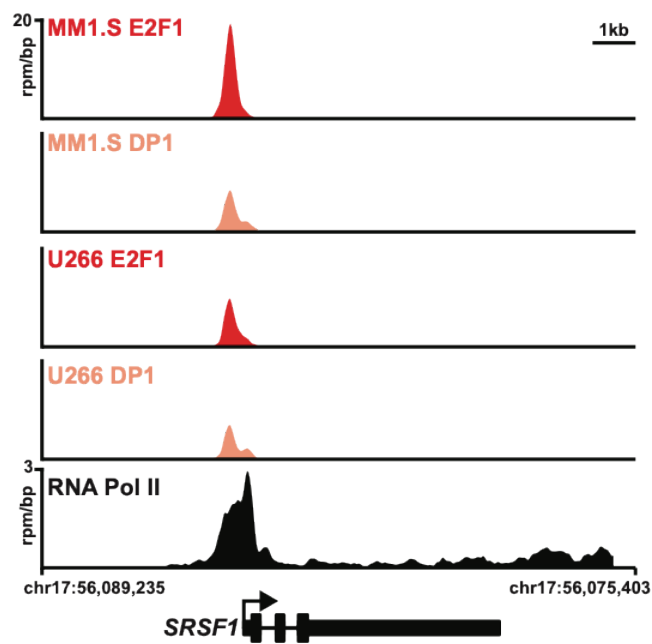

B

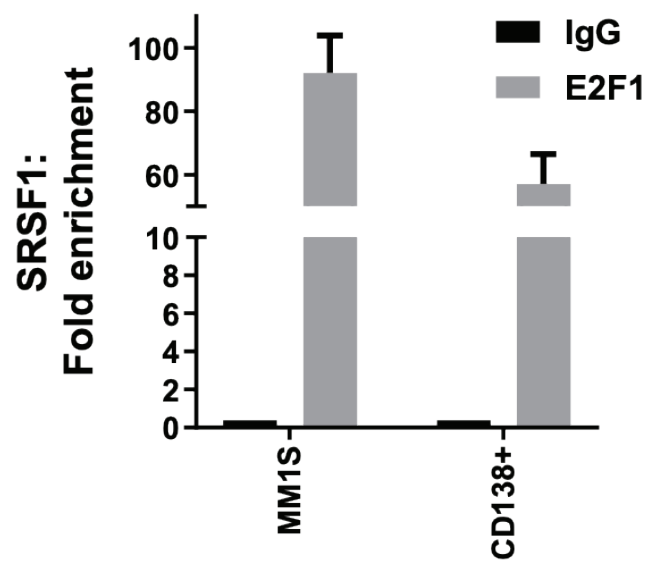

C

DP1/E2F binding disruption

0 10 20 RK-19 ( $\mu$ M)

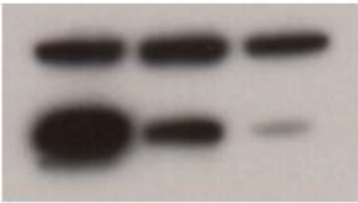

Tubulin  
SRSF1

Supplement: Supplementary file 7 — Supplementary Figure 6 [file 41408_2022_759_MOESM7_ESM.pdf]
